# Patient Characteristics and Clinical Outcomes of Acute Pyelonephritis Treated in Mayo Clinic's Hospital-at-Home Program

**DOI:** 10.1093/ofid/ofaf748

**Published:** 2026-01-15

**Authors:** Cesar A Gomez-Cabello, Igor Dumic, Michael J Maniaci, Margaret R Paulson, Aryan Shiari, Leah W Webster, Jeni McGrew, Ariana Genovese, Bernardo Collaco, Maissa Trabilsy, Antonio J Forte, Wendelyn Bosch

**Affiliations:** Division of Plastic Surgery, Mayo Clinic, Jacksonville, Florida, USA; Department of Hospital Medicine, Mayo Clinic Health System, Eau Claire, Wisconsin, USA; Division of Hospital Internal Medicine, Mayo Clinic, Jacksonville, Florida, USA; Department of Hospital Medicine, Mayo Clinic Health System, Eau Claire, Wisconsin, USA; Department of Pulmonary and Critical Care Medicine, Mayo Clinic Health System, Eau Claire, Wisconsin, USA; Department of Pharmacy, Mayo Clinic, Jacksonville, Florida, USA; Division of Hospital Internal Medicine, Mayo Clinic, Phoenix, Arizona, USA; Division of Plastic Surgery, Mayo Clinic, Jacksonville, Florida, USA; Division of Plastic Surgery, Mayo Clinic, Jacksonville, Florida, USA; Division of Plastic Surgery, Mayo Clinic, Jacksonville, Florida, USA; Division of Plastic Surgery, Mayo Clinic, Jacksonville, Florida, USA; Division of Infectious Diseases, Mayo Clinic, Jacksonville, Florida, USA

**Keywords:** bacteremia, hospital-at-home, pyelonephritis, readmission, sepsis

## Abstract

**Background:**

Outcomes of patients with acute pyelonephritis (AP) treated in a hospital-at-home setting have not been comprehensively evaluated in the United States.

**Methods:**

We performed a multicenter, retrospective cohort study of adults diagnosed with and managed for AP in Mayo Clinic's Advanced Care at Home (ACH) program between July 2020 and January 2025. Collected data included demographics, Charlson Comorbidity Index (CCI), genitourinary comorbidities, severity of illness (SOI), and risk of mortality (ROM) scores, as well as pyelonephritis-related complications. Outcomes included length of stay (LOS), escalation of care, and 30-day postdischarge emergency department (ED) visits, readmissions, and mortality.

**Results:**

A total of 165 patients met inclusion criteria. Median age was 67 years; SOI scores were moderate in 33.3%, major in 52.1%, and extreme in 8.5%. ROM scores were moderate in 30.3%, major in 38.2%, and extreme in 6.7%. Median CCI score was 5, and all patients had preexisting genitourinary conditions. On admission, 30.9% met sepsis criteria, acute kidney injury was present in 47.3%, and bacteremia developed in 33.3%. Median LOS in the ACH program was 3.1 days. Only 4.8% required escalation to a brick-and-mortar hospital. Readmission occurred in 17.0%, and 4.8% had ED visits. No in-program deaths occurred.

**Conclusions:**

This multicenter retrospective study shows that AP, including cases with high illness severity and complex comorbidities, can be managed safely and effectively in a hospital-at-home setting with careful patient selection and robust infrastructure to support timely escalation when needed.

Acute pyelonephritis (AP) is most often managed in the outpatient setting, while approximately 15%–20% (an estimated 100 000 patients) require hospitalization [1–4]. Hospitalization is typically reserved for patients who are hemodynamically unstable, unable to receive or failed oral antimicrobials, require intravenous analgesics or fluids, have complications, are at high risk for clinical deterioration, or are pregnant [1, 2]. Hospitalized patients with AP in the United States (US) have an average length of stay (LOS) between 2.8 and 4 days with annual cost estimated at $2.14 billion [4–6]. A large analysis of US hospitalization in 2003 found that the in-hospital mortality rate for patients with AP is approximately 0.8%, is higher among men (1.65%) compared to women (0.73%), and increases with advancing age and comorbid conditions [6–8]. More recent studies from Spain and France showed mortality in AP to be higher—around 6% [9, 10].

Over the past decade, hospital capacity constraints and a growing elderly population led to the emergence of hospital-at-home (HaH) programs across the US. Multiple randomized controlled trials and observational studies have demonstrated that HaH programs provide comparable or better clinical outcomes (30-day mortality, readmission, and adverse events) than traditional brick-and-mortar (BaM) hospitals, while increasing patient satisfaction [11–18]. Although acute urologic conditions can be managed safely in a HaH setting [19] and prior cohorts that evaluated HaH outcomes included some patients with AP [11], there is a lack of research on detailed patients' characteristics and outcomes of patients with AP managed in a HaH program. Our study aim is to describe the characteristics and clinical outcomes of patients admitted for AP to a multicenter HaH program located in 3 US states from July 2020 to January 2025.

## METHODS

### Study Setting

Mayo Clinic's HaH program, known as Advanced Care at Home (ACH), uses a virtual-hybrid model which combines a centralized virtual command center with in-person home care. Patients with acute medical conditions are eligible for ACH if they are clinically stable, have a safe home environment, pass a social screening, and meet payer eligibility. Clinical stability is defined by the patient having (1) normal or noncritical abnormal vital signs and (2) normal or baseline mental status without the need or presence of the following: vasopressor support, high-flow oxygen therapy >5 L/minute, new noninvasive positive pressure ventilation, intensive care unit (ICU) level of care, urgent or emergent surgery or invasive procedure, and multiorgan failure. Those who meet inpatient criteria and are eligible for ACH are transferred directly to our program from the emergency department (ED) or from the BaM hospital unit. Upon admission, patients are transported home and receive twice-daily in-person visits by a nurse or paramedic and once-daily in-person or virtual visit by an advanced practice provider, carrying out treatment care plans instituted from virtual hospital medicine physicians. The care team delivers hospital-level interventions, including intravenous antibiotics, diuretics, steroids, fluids, oxygen therapy, nebulized treatments, wound care, and laboratory and imaging services as needed. Inpatient pharmacists with specialized expertise in HaH play a pivotal role in ensuring safe and effective medication management. They oversee the medication history and reconciliation process, verify prescribed medications, and make clinical adjustments tailored to the unique requirements of HaH care delivery as well as each patient's unique needs.

Antimicrobial stewardship experts are embedded in hospital practice, including HaH, and alert HaH attending physicians of recommended changes based on culture and susceptibility data. Daily HaH multidisciplinary rounds include a case manager for discharge planning and to anticipate if intravenous antibiotics will be needed once patient completes their hospitalization. Remote monitoring devices, such as pulse oximeters, blood pressure cuffs, thermometers, and oxygen concentrators, are deployed to allow real-time tracking of vital signs and clinical status. Rapid escalation protocols are in place at each site to ensure patient safety in the event of clinical deterioration. The command center coordinates all aspects of care, including 24/7 physician availability, emergency return-to-hospital pathways, and escalation of care for advanced imaging or procedures. Social work and care coordination are integrated to address psychosocial needs and ensure smooth care transitions. Outcomes from the Mayo Clinic's program show low in-program and 30-day mortality, low readmission rates, and safe management of acute conditions in both urban and rural settings, as well as high patient satisfaction [17, 19–21]. Since the inception of Mayo Clinic's ACH program in July 2020, >7000 patients have been treated across our sites.

### Study Design and Patient Population

We conducted a retrospective cohort study of patients diagnosed and managed with AP in ACH from July 2020 through January 2025. This is a descriptive outcome study that included patients with AP from all 3 ACH sites: Mayo Clinic in Florida, a 304-bed urban academic medical center in Jacksonville, Florida; Mayo Clinic Health System Eau Claire, a 304-bed community teaching hospital in Eau Claire, Wisconsin; and Mayo Clinic in Arizona, a 368-bed academic medical center in Phoenix, Arizona. Inclusion criteria consisted of adult patients initially identified by registered diagnoses of “upper urinary tract infection,” “kidney and urinary tract infection,” or “other kidney and urinary tract diagnoses,” subsequently confirmed as pyelonephritis. A diagnosis of AP was made by a hospital medicine physician or advanced practice provider, based on clinical presentation (fever, flank pain, and urinary symptoms) and laboratory findings (pyuria, bacteriuria, leukocytosis, and elevated inflammatory markers). In 75% of patients, the diagnosis was further supported by characteristic imaging findings on computed tomography with evidence supportive of pyelonephritis. We excluded patients managed for lower urinary tract infection (UTI) who did not meet the above criteria for pyelonephritis. The study protocol was approved by the Institutional Review Board of Mayo Clinic (Protocol Number 24-001934), and all procedures adhered strictly to ethical guidelines concerning patient confidentiality and data protection.

### Data Collection

Baseline data were systematically extracted from the electronic health record (EHR) by trained reviewers. Sociodemographic characteristics, Charlson Comorbidity Index (CCI) score [22], and genitourinary comorbidities were collected. Clinical characteristics at time of hospital admission included severity of illness (SOI) and risk of mortality (ROM) scores (minor, moderate, major, and extreme) based on the All Patient Refined Diagnosis-Related Groups [23, 24], presence of sepsis [25], acute kidney injury (AKI), bacteremia, bacteriuria, imaging findings, and pyelonephritis-related complications. Treatment details included antimicrobial agents used with duration of therapy, administration of intravenous fluids (bolus or continuous infusion), and adjunctive medications, including antiemetics and analgesics. The patient pathway to the ACH program was categorized as acute substitution (admission to ACH directly from ED) or transfer from BaM hospital unit. Our clinical outcomes data consisted of inpatient BaM and ACH LOS; frequency and reason for BaM escalation of care; postdischarge 30-day ED visits; hospital readmission (BaM and/or ACH) at 7, 14, and 30 days; and 30-day mortality.

### Statistical Analysis

Descriptive statistics were calculated to summarize patient demographics, clinical characteristics, treatment variables, and clinical outcomes. Continuous variables are presented as median with interquartile range (IQR [Q1, Q3]), and categorical variables as frequencies and percentages.

## RESULTS

### Baseline Characteristics

Between 6 July 2020 and 31 January 2025, 165 patients met criteria for AP and were included in the analysis. The median age was 67 years (IQR, 56–75 years), with 96 individuals (58.2%) identified as female and 140 (84.8%) identifying as White. At the time of enrollment to ACH, SOI scores were classified as minor in 9 patients (5.5%), moderate in 55 (33.3%), major in 86 (52.1%), extreme in 14 (8.5%), and missing for 1 patient (0.6%). ROM scores were minor in 40 patients (24.2%), moderate in 50 (30.3%), major in 63 (38.2%), extreme in 11 (6.7%), and missing in 1 (0.6%) case. The median CCI score was 5 (IQR, 2–7). All patients had preexisting genitourinary conditions. A history of recurrent UTIs was documented in 112 patients (67.9%), and chronic kidney disease was present in 84 patients (50.9%). A total of 47 patients (28.5%) had a history of renal transplantation and 16 (9.7%) were receiving chronic dialysis. Demographic and baseline clinical characteristics are summarized in Table 1.

**Table 1.** Demographic Characteristics and Comorbidities

Patient Demographics	No. (%) (N = 165)
Age, y	
Mean	63.25
Median (IQR)	67 (56–75)
Sex	
Female	96 (58)
Male	69 (42)
Hospital-at-home site	
Florida	93 (56)
Arizona	41 (25)
Wisconsin	31 (19)
Race	
Non-Hispanic White	140 (85)
Black or African American	15 (9)
Asian	8 (5)
Unknown	1 (1)
Severity of illness^a^	
Minor	9 (5)
Moderate	55 (33)
Major	86 (52)
Extreme	14 (8)
Risk of mortality^a^	
Minor	40 (24)
Moderate	50 (30)
Major	63 (38)
Extreme	11 (7)
CCI score	
Mean	4.68
Median (IQR)	5 (2–7)
Genitourinary conditions	
Recurrent UTI	112 (68)
Chronic kidney disease	84 (51)
Renal transplant	47 (28)
Ureteral stent	47 (28)
Nephrolithiasis	39 (24)
Indwelling or suprapubic catheter	28 (17)
Bladder cancer	22 (13)
Prostate cancer	19 (12)
Ureteral stricture	18 (11)
Ileal conduit	18 (11)
Hemodialysis	16 (10)
Intermittent urinary catheter	16 (10)
Neurogenic bladder	11 (7)
TURP	11 (7)
Prior lithotripsy	10 (6)
Urethral stent	9 (5)
Pelvic floor dysfunction	8 (5)

N = number of participants; n = number of participants in each category; % = (n/number of participants with available data) × 100.

Abbreviations: CCI, Charlson Comorbidity Index; IQR, interquartile range; TURP, transurethral resection of the prostate; UTI, urinary tract infection.

^a^Severity of illness score and risk of mortality score based on All Patient Refined Diagnosis-Related Groups (APR-DRG).

### Clinical Characteristics and Outcomes

Fifty-one patients (30.9%) met sepsis criteria on admission, including 6 (3.6%) with severe sepsis and 5 (3.0%) with septic shock. AKI developed in 78 (47.3%), and bacteremia was documented in 55 (33.3%). Imaging was obtained in 130 (78.8%) via computed tomography and 65 (39.4%) via ultrasound. Of these, 32 (19.4%) had imaging findings of pyelonephritis complications. A summary of the clinical presentations is presented in Table 2. Of the patients who presented with sepsis, 13 (25.4%) were directly admitted to ACH as acute substitution from the ED, while 38 (74.5%) were stabilized initially in the BaM hospital before being transferred to ACH.

**Table 2.** Clinical Presentations and Complications

Clinical Presentation	No. (%) (N = 165)
Acute kidney injury	78 (47)
Bacteremia	55 (33)
Sepsis	51 (31)
Severe sepsis	6 (4)
Septic shock	5 (3)
Complications on imaging	(n = 32 [19%])
Hydronephrosis	10 (31)
Hydroureteronephrosis	9 (28)
Abscess	4 (12)
Perinephric fluid	3 (9)
Hemorrhage	2 (6)
Renal calculus	1 (3.1)
Metastatic disease	1 (3.1)
Cortical scarring	1 (3.1)
Cystitis	1 (3.1)
Striated nephrogram	1 (3.1)
Stent migration	1 (3.1)

N = number of participants; n = number of participants in each category; % = (n/number of participants with available data) × 100. Sepsis, severe sepsis, and septic shock were defined according to Sepsis-3 criteria.

**Figure 1.**
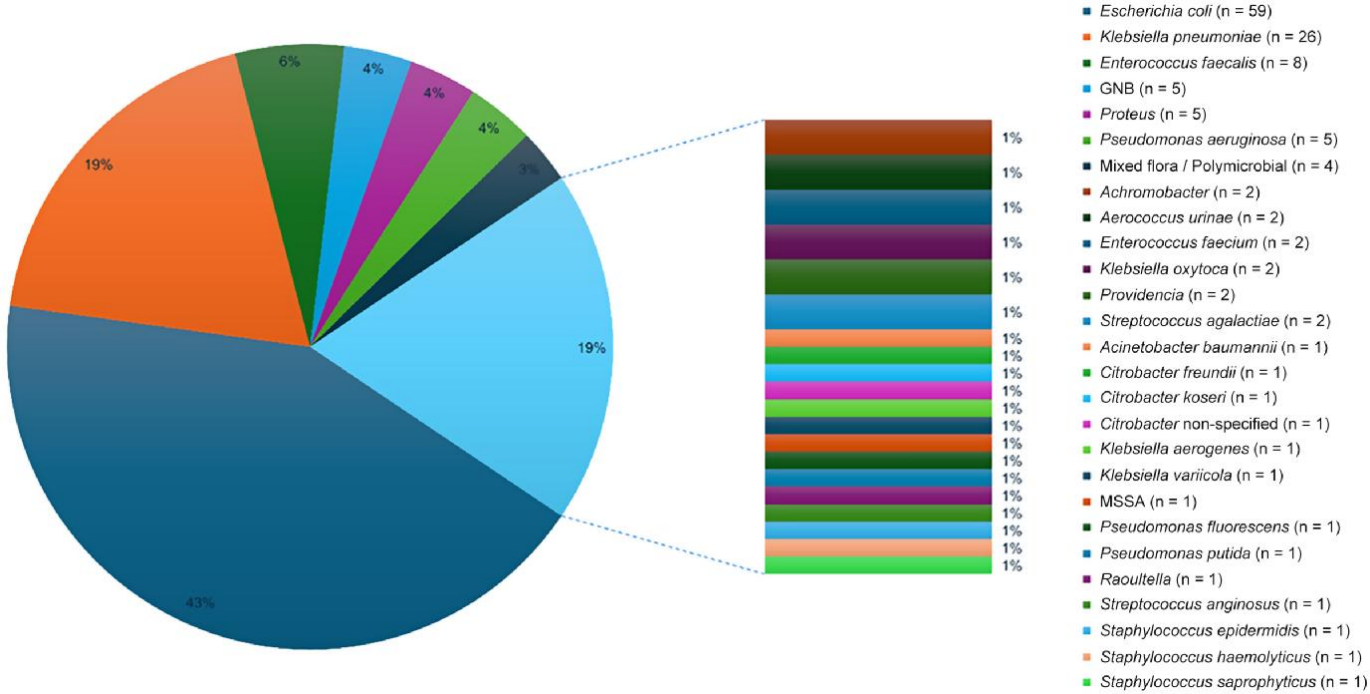
Various pathogens isolated from urine cultures. Abbreviations GNB, gram-negative bacteria; MSSA, methicillin-susceptible *Staphylococcus aureus*.

Urine cultures were obtained in 123 (74.5%) of the patients, of which 70 (56.9% of cultures performed; 42.4% of the overall cohort) yielded significant microbial growth. A total of 27 pathogens were isolated, with *Escherichia coli* being the most common (n = 59 [84.3%]), followed by *Klebsiella pneumoniae* (n = 26 [21%]). The full list of isolated microorganisms is shown in Figure 1.

**Figure 2.**
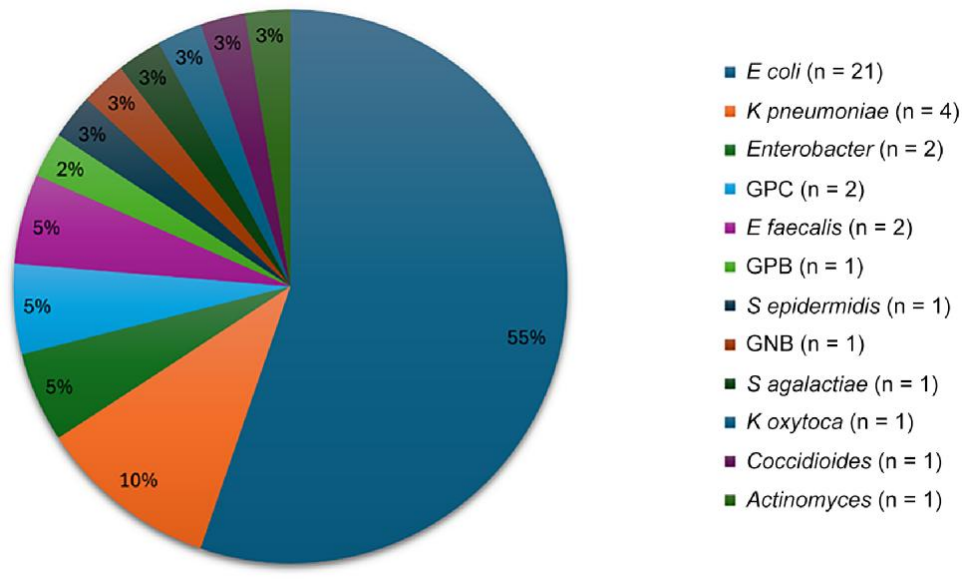
Various pathogens isolated from blood cultures. Abbreviations: GNB, gram-negative bacteria; GPB, gram-positive bacilli; GPC, gram-positive cocci.

Fifty-four patients (33%) were diagnosed with bacteremia and 1 patient (0.6%) with fungemia, with 12 different isolated pathogens in 37 patients by the time of HaH stay. In patients with AP who had both bacteremia and positive urine cultures, the results of the 2 cultures were concordant. However, not all patients with bacteremia had positive urine cultures, and vice versa. *Escherichia coli* was the most common, identified in 21 (55.3%) patients, and was followed by *K pneumoniae*, isolated in 4 (10.5%) patients. A comprehensive presentation of blood culture results in our cohort is presented in Figure 2.

Regarding ACH logistics and management, 55 patients (33.3%) were admitted as acute substitutions from ED and 110 (66.7%) from a BaM hospital unit. The median total LOS was 4.2 days (IQR, 3.1–6.3 days), comprising a median BaM LOS of 1.0 days (IQR, 0.58–1.8 days) prior to ACH transfer and a median ACH LOS of 3.1 days (range, 2.0–4.9 days). A total of 63 patients (38.2%) required antimicrobial regimen changes based on final microbial results once they were transferred to ACH. Of the 63 patients (38%) who had a change in their antimicrobial regimen while at ACH, 10 patients (6%) had their therapy escalated, while in the remaining 32%, the antibiotics were de-escalated based on culture results. Among the 10 patients who required escalation, 5 had infections caused by extended-spectrum β-lactamase (ESBL)–producing *E coli*, leading to a change in therapy from a third-generation cephalosporin or piperacillin-tazobactam to ertapenem. In 2 patients, cultures grew ESBL-producing *K pneumoniae*, necessitating escalation from ceftriaxone to ertapenem. In 1 patient, ceftriaxone was changed to cefepime after cultures grew *Enterobacter cloacae* complex. The remaining 2 patients had infections caused by *Achromobacter xylosoxidans* and *Providencia* species, requiring changes from ceftriaxone to levofloxacin and cefepime, respectively. The median duration of antibiotic therapy was 5 days (range, 1–36 days). Intravenous fluids were given as boluses in 108 (65.5%) patients and as a continuous infusion in 22 (13.3%). Antiemetics were administered to 104 (63.0%) and analgesics to 153 (92.7%). Table 3 summarizes treatments received by patients while hospitalized at home.

**Table 3.** Various Treatment Used at Mayo Clinic's Hospital-at-Home Program for Patients Admitted With Acute Pyelonephritis

Hospital-at-Home Treatments	No. (%) (N = 165)
Intravenous antibiotics	165 (100)
Antibiotic days, mean	5
Empiric antibiotic change after culture results	63 (38)
Escalation	53 (32)
De-escalation	10 (6)
Intravenous fluids	132 (80)
Bolus	108 (65)
Continuous	22 (13)
Both	2 (1)
None	33 (20)
Analgesic use	153 (93)
Antiemetic use	104 (63)

N = number of participants; n = number of participants in each category; % = (n/number of participants with available data) × 100.

Of the 165 patients, only 8 (4.8%) were escalated back to the BaM hospital: 2 (25.0%) for lack of improvement, 2 (25.0%) for suprapubic catheter complications, 1 (12.5%) for procedure-related complications, 1 (12.5%) for worsening AKI and bradycardia, 1 (12.5%) due to lack of caregiver support, and 1 (12.5%) for a nephrostomy tube placement, who then remained in BaM for the duration of hospitalization. Twenty-eight (17.0%) patients were readmitted within 30 days from ACH discharge. Of these, 15 (53.6%) were readmitted within 7 days, 5 (17.9%) within 14 days, and 8 (28.6%) within 30 days. Among these patients, 19 (68%) required admission to the BaM hospital, while 6 (21%) were readmitted to ACH, and 3 (11%) were readmitted in BaM, then transitioned back to ACH. From the total population, 8 (4.8%) patients visited the ED within 30 days of ACH discharge, 6 of them due to pyelonephritis-related reasons, such as ongoing dysuria (n = 3), abdominal pain (n = 2), hematuria (n = 1), and worsening peripheral edema (n = 1). The 2 remaining patient visits were for unrelated causes such as dizziness (n = 1) and dislodged urinary catheter (n = 1). There were no in-program or 30-day postdischarge mortalities.

## DISCUSSION

This is the first US, multicenter, retrospective cohort study, describing in detail the characteristics, care, and outcomes of patients admitted to a HaH program for management of AP. Prior studies from the US have identified AP as one of the common diagnoses that can be managed in HaH [9]. However, the outcome data in these studies were aggregated with other infectious and noninfectious conditions, limiting insights specific to AP. The novelty of our study lies in providing a detailed characterization of patients with AP and focusing exclusively on AP-related complications and outcomes. Our observational study found that most patients admitted to the HaH program were complex, reflected by 85% having a moderate to major SOI score, 68% having a moderate to major ROM score, and 100% with underlying genitourinary comorbid conditions. We had favorable clinical outcomes with low escalation rates to BaM (4.8%), 30-day ED visits (4.8%), and no 30-day mortality. To our knowledge, Regalado and colleagues' study from Spain in 2006 is the only one that describes the management of AP in a HaH program [26]. This study included 369 patients with AP without septic shock or complications of pyelonephritis (AKI, bacteremia, or urinary obstruction), making it quite different than our study cohort. This highlights the evolution of HaH from a simple model caring for low-acuity patients to one capable of safely caring for severely ill, highly complex patients.

Recent US studies have shown a rising number of hospitalizations for complicated UTIs (cUTIs) with associated increase in healthcare cost. For instance, one study from 1998 to 2011 calculated the financial impact of approximately 400 000 hospitalizations related to cUTI to cost $2.8 billion [27]. Subsequently, a retrospective analysis of the PharmMetrics Plus database from 2013 to 2017 evaluated 104 866 adult hospitalizations for cUTI and found the median cost of initial admission to be $9441 for a median LOS of 4 days. Among these inpatients, 2.3% experienced a subsequent readmission, which leads to even further costs for a genitourinary condition [28]. Another study using the Premier Healthcare Database from 2013 to 2018 analyzed 187 789 hospitalized cUTI patients and although 18.9% were considered low-acuity (no sepsis or systemic symptoms) hospitalizations, it led to a substantial median cost of $5575 for a median LOS of 3 days [29]. These findings underscore the substantial economic burden associated with this diagnosis and the need for equally efficacious alternative treatment settings that reduce the financial impact. In this context, the HaH model may represent a viable option as it becomes more widely adopted in the US.

A cross-sectional study by Gottlieb et al examined adult ED patients in the US diagnosed with pyelonephritis between 1 January 2016 and 31 December 2023 [30]. Among 205 526 173 total ED encounters, pyelonephritis was present in 1 044 742 cases (0.5%), and 33.4% of these patients were admitted to the hospital. The study did not report mortality rates. In specific populations, such as pregnant and postpartum women, a recent study from Duke University analyzed 32 850 hospital admissions for pyelonephritis [31]. The majority of these cases (89%) occurred during the antepartum period; however, the rates of severe maternal morbidity were higher in the postpartum group than in the antepartum group. The relative risk for composite severe maternal morbidity in postpartum hospitalizations was 4.65% and was primarily driven by sepsis. In our HaH cohort, we did not include any pregnant or postpartum patients. This was not due to intentional exclusion but rather because such patients were not represented by chance.

In our cohort, measurable outcomes, including readmission rates, LOS, and in-program mortality, were favorable and similar to the metrics reported in other US studies of BaM hospitalizations for cUTI [27–29]. The only HaH study that we can closely compare our pyelonephritis patient outcomes to is to Regalado et al from Spain [26]. In their study, the average LOS was 5 days, with intravenous antibiotics administered for 3 days. The readmission rate was low at 4%, and it was mainly due to hypotension, vomiting, pain, fever, ultrasound findings, or patient request. In our study, the duration of antibiotic treatment and LOS in ACH was comparable but the readmission rate was higher, and as Regalado et al did not report on SOI or ROM, it would be impossible to make a direct comparison. In the US, Levine and colleagues' randomized controlled trial of HaH adults with a mixed admission diagnosis reported a 30-day readmission rate of 7% [16], while a pragmatic ACH randomized trial from Mayo Clinic found an unplanned 30-day readmission rate to be 15% [17]. However, these studies did not report ROM or SOI in their cohorts, which also limits the direct comparison with our group. One possible explanation for the higher 17% readmission rate in our cohort is the inclusion of a sicker patient population at baseline, including individuals with urogenital malignancies and kidney transplant recipients. Conversely, data from de Stampa et al in France indicate a 27.8% readmission rate among older adults (>75 years) hospitalized for cancer care [32], while in another monocentric French HaH study, Barré et al reported a 16% 30-day readmission rate [33]. Observational data from Lim et al in Australia reported a 30-day readmission rate of 11.9% [34], while Yehoshua et al in Israel found HaH 30-day readmission rates of approximately 13.5% for other common infections such as pneumonia and cellulitis [35].

For comparison, a study from the Mayo Clinic's ACH program involving 173 patients with COVID-19 pneumonia, of which 43.3% had extreme SOI and 46.2% had extreme ROM, documented escalation of care in 7.5% and 30-day readmission of 9.2% [14]. In our cohort, the average CCI score was 5, indicating a high comorbidity burden. This level of comorbidity is associated with a significantly increased risk of both short- and long-term mortality compared to patients with lower scores, emphasizing the complexity of the patients we safely managed at home [22]. **S**epsis was present in 30% of patients; 33% had bacteremia and 47% experienced AKI—all markers of severe pyelonephritis. Taken together, this acuity profile is incompatible with management in an outpatient setting. Active sepsis, bacteremia, and AKI in the context of major/extreme SOI/ROM and a mean CCI score of 5 necessitate frequent assessment, rapid rescue capability, and multidisciplinary coordination (ie, an inpatient level of care). ACH delivers those inpatient capabilities in the home through protocolized sepsis pathways, high-frequency reassessment. and remote patient monitoring with real-time alerts, same-day labs/therapy adjustments, and on-demand community paramedic response under physician oversight. The absence of deaths in our current study and the very low escalation rate therefore reflect safe hospital-level care at home, not selection of low-risk patients for convenience. In fact, our ability to safely manage a significant percentage of these high-risk patients at home also demonstrates the effectiveness of careful patient selection and appropriate ACH monitoring. This stands in stark contrast to Meersohn and colleagues' study of 28 patients in Israel with bacteremia, where 30% required escalation to higher levels of care and 2 patients died [36].

The BaM escalation rate in our cohort was 4.8%, significantly lower than the 15% reported in a recent clinical trial from the same institution [17]. This difference likely reflects the careful patient selection in our study, whereas patients in the other trial were randomized. In a scoping review analyzing reasons for escalation that included 23 papers, it was identified that HaH programs treating infectious diseases had the highest escalation rates, with an overall range between 3.6% and 21.4%. Moreover, the authors identified that the most common reasons for escalation were a lack of treatment response, exacerbation of the basal disease, scheduled procedures, and caregiver-related reasons [37]. Our study results mirror those described in that scoping review. Among the 8 ACH patients escalated back to the BaM hospital, reasons included the need for advanced procedures such as renal abscess drainage, urethral stent placement for hydronephrosis, lack of treatment response, and one case of progressive weakness combined with loss of caregiver support, creating an unsafe home environment and high fall risk. Importantly, all 8 ACH patients who required escalation of care were recognized promptly, transferred efficiently, and recovered fully; there were no deaths related to ACH care. None of the escalated patients required ICU level of care. This underscores the importance of having a robust and safe rapid response system and escalation protocols in every HaH program to ensure timely and effective transfer when clinical deterioration occurs.

Our study has many strengths. To our knowledge, this is the first study analyzing specifically patients treated for AP in a HaH program in the last 2 decades and is the only study from the US that includes patients with AP complicated by sepsis, bacteremia, AKI, and obstructive uropathy. By evaluating 165 adults with pyelonephritis from 3 geographically distinct Mayo Clinic ACH sites—a tertiary academic medical center in Florida, a rural community teaching hospital in Wisconsin, and a tertiary care center in Arizona—we not only increased the generalizability of our findings but also captured the real-world complexity of urologic infections managed in a HaH setting. Our integration of advanced digital health technologies and coordination by a multidisciplinary team underscores the feasibility of delivering complex care at home. Nevertheless, our reliance on EHR documentation and the retrospective nature of the study carry the inherent risks of incomplete capture of data and limitation. Further prospective studies analyzing predictors for readmission in this population are paramount to improving the management of these patients at home. Moreover, a comprehensive study with a control group of patients who remained at a BaM hospital would help better understand unmeasured factors that could influence readmission risk in HaH patient populations, including caregiver availability, digital literacy, and social barriers.

## CONCLUSIONS

This 165-patient multicenter, retrospective cohort demonstrates that adults with AP, including those with a high severity of illness and complexity, can be managed safely and effectively in a HaH setting. Given our patients' high-acuity profile (sepsis, AKI, bacteremia, high SOI and ROM), these patients were not candidates for outpatient treatment and required inpatient-level care. Our outcomes of no deaths and a very low escalation rate to BaM demonstrate that ACH can safely deliver inpatient pyelonephritis care in the home. These findings suggest that, with rigorous patient selection criteria, robust remote monitoring, and safe and efficient rapid escalation pathways, HaH models can extend beyond traditional low-acuity conditions to support complex urologic infections. Prospective, controlled studies are warranted to compare outcomes and cost-effectiveness against conventional inpatient care, and to identify predictors of readmission with the goal of optimizing care pathways for patients with pyelonephritis.
